# SUPPORTING FAMILY CAREGIVERS OF OLDER PATIENTS WITH DEMENTIA: A PSYCHOEDUCATIONAL MODEL IN SHANGHAI

**DOI:** 10.1093/geroni/igad104.1597

**Published:** 2023-12-21

## Abstract

In China, where community-based health and social supports designated for persons living with dementia (PLWD) and caregivers are few and scarce, doctors at the neurological and geropsychiatric clinics and hospitals are expected to provide psychoeducation programs for family caregivers. Geropsychiatric doctors, in particular, championed the efforts to provide counseling, education, and support to family caregivers when they are not able to handle the psychological and behavioral problems of PLWD. This case study examines the preliminary efficacy of the efforts of the geropsychiatric unit in the largest mental health center in Shanghai to provide education, empowerment, and supports to family caregivers of dementia. A total of 40 dementia caregivers were interviewed to identify their unmet service needs when their PLWD visited the clinic or were admitted to the inpatient unit. Most caregivers reported a knowledge deficit in dementia treatment and care and expected to receive training and support to improve their understanding and skills. In line with their needs, the researcher developed a program facilitating the dialogue between medical staff and caregivers. The dialogue occurs once a week, includes the medical staff and up to 6 family caregivers, and often last for 90 minutes. Each week focuses on a different topic covering the medical procedures, diagnosis and treatment, person-centered care for the PLWD, self-care for the caregiver, and transition care needed for community living. Caregivers who received these sessions indicated great satisfaction with this program, increased confidence, and decreased worries and anxieties.

